# A Fatal Case of Neuroinvasive West Nile Virus Infection in an Immunocompromised Host

**DOI:** 10.51894/001c.5967

**Published:** 2017-08-24

**Authors:** Justin Allen, Jennifer Conard, Michael Wang

**Affiliations:** 1 Lakeland Health Internal Medicine Resident, PGY 3, Saint Joseph, M; 2 Lakeland Health Internal Medicine Resident, PGY 3, Saint Joseph, MI; 3 Lakeland Health IM Residency Program Director and Attending Physician Infectious Diseases, Saint Joseph, MI

**Keywords:** neuroinvasive disease, immunocompromised host, west nile encephalitis

## Abstract

The neuroinvasive form of West Nile disease is an uncommon manifestation of the viral infection. To date, documented cases in Michigan of neuroinvasive decompensation from this virus have been rare. Evaluation requires a broad differential diagnosis and treatment options are still quite limited. Objective evaluations entailing physical exam and radiographic and laboratory changes are nonspecific. Serologic testing of cerebrospinal fluid by enzyme immunoassay remains the gold standard for diagnosis. However, IgM antibodies typically do not develop until after the fourth to seventh day of symptom onset. This retrospective case report presents an immunocompromised male patient in his mid-70s in whom neuroinvasive West Nile virus was diagnosed postmortem. All information was obtained from the patient’s electronic health record. This patient’s immunocompromised state at the time of West Nile exposure made him more susceptible to neuroinvasive disease progression and ultimately influenced the outcome. Prior to withdrawing care, the patient was treated for methicillin sensitive staphylococcus aureus (MSSA) cellulitis and Type 1 Herpes Simplex virus. In this case, neuroinvasive West Nile virus was a less likely diagnosis given the patient’s physical exam findings and the context of more likely alternative explanations for his cognitive decline. Treatment options for neuroinvasive forms of West Nile virus are still supportive and would not have altered the patient’s hospital course. This case report demonstrates that clinicians must maintain an ongoing index of suspicion for infection in warmer climates where West Nile is becoming more prevalent. Given some patients’ obscure physical exam findings and radiographic imaging results, a thorough history with laboratory conformation is required for a more conclusive diagnosis

## INTRODUCTION

West Nile virus (WNV) typically presents as an asymptomatic febrile illness. In 1% of patients, WNV can progress to aseptic meningitis, encephalitis or poliomyelitis syndrome.^1^ The virus is transmitted to humans following a bite from an infected mosquito that acquires the pathogen after feeding on a host, typically a bird. Person-to-person transmissions have also been observed following transfusion of blood products, organ transplantation, in hemodialysis patients, and through intrauterine, percutaneous, respiratory aerosol or breastfeeding exposure.^1^ Patients with hematologic malignancies are also at an increased risk for the neuroinvasive form of West Nile disease and have a poorer prognosis.^2^

Serologic testing of cerebrospinal fluid (CSF) by enzyme immunoassay remains the gold standard for WNV diagnosis. IgM antibodies typically develop by the fourth to seventh day after onset of symptoms. Detection of WNV IgM in CSF is diagnostic of neuroinvasive disease. Since WNV IgM antibodies do not passively diffuse across the blood-brain barrier, CSF containing WNV IgM is needed to confirm intrathecal (spinal canal) penetration. Diagnosis can also be confirmed by WNV plaque reduction neutralization assay laboratory test (PRNT). WNV IgM antibodies, detected through enzyme-linked immunosorbent assay (ELISA), may persist after infection for twelve to sixteen months in the serum and seven months in the CSF.^2^

In immunosuppressed patients, polymerase chain reaction (PCR) testing has a higher sensitivity than either PRNT or ELISA assay tests because these patient’s may not have yet mounted sufficient antibodies to the virus.^1^ The incidence of MRI abnormalities is variable and pathologic findings in WNV encephalitis, such as hyperintensity or signal changes on magnetic resonance imaging (MRI), are nonspecific.

### Case Report

This patient was a male in his mid-70’s with a contributing past medical history of colon cancer status-post resection and colostomy, basal cell carcinoma, Lyme disease treated with doxycycline and chronic lymphocytic leukemia (CLL). His CLL had been diagnosed four years before and he was currently treated with chlorambucil, rituximab, and bendamustine. Following a chemotherapy holiday, he had been started on ibrutinib three months prior to presentation. In September 2016, the patient presented to the emergency department at the recommendation of his oncologist for evaluation of his fever, fatigue, weakness, delirium and a left lower extremity lesion. The lesion was the result of a punch biopsy that had been performed three days earlier. 

He was initially admitted to the general medical hospital floor for sepsis secondary to purulent cellulitis at the biopsy site. Cultures of the site were obtained and he was started on a combination of piperacillin-tazobactam and vancomycin. During his first hospital night, he experienced an episode of vomiting after which he developed acute respiratory failure, ultimately requiring his intubation and mechanical ventilation, as well as transfer to the critical care unit (CCU). A chest x-ray completed after intubation confirmed a right interstitial infiltrate.

In the CCU, the patient was started on norepinephrine for septic shock. A superficial culture of the lower extremity wound confirmed methicillin sensitive staphylococcus aureus (MSSA). The Infectious Disease service was consulted for assistance in his management. Since this patient appeared to be in septic shock from cellulitis, vancomycin was discontinued in favor of clindamycin for its antitoxin effects. Anidulafungin was also added for broad spectrum coverage for possible fungemia (i.e. presence of fungi or yeast in the blood). His blood cultures and 1,3 β D glucan level, often elevated in disseminated fungal infections, were negative.  However, an endotracheal tube aspiration specimen was remarkable for yeast.  Culture sensitivities for MSSA were sensitive to piperacillin-tazobactam, therefore clindamycin and anidulafungin were discontinued.

As the patient became more hemodynamically stable, vasopressors and sedation medication doses were tapered in an attempt to wean him from mechanical ventilation. However, he remained comatose with intermittent fevers. An MRI of the brain revealed possible right thalamic ischemic changes. The Neurology service was consulted due to his persistent neurologic dysfunction. A lumbar puncture was performed demonstrating both an elevated CSF opening pressure and protein level with a predominance of lymphocytes.

On physical exam, a herpetic-appearing lesion on his lower lip was also noted. In the context of his immunosuppression, acyclovir was initiated since there was concern for possible development of Herpes Simplex viral (HSV-1) encephalitis or meningitis. The lip ulceration cultured positive for HSV-1 by PCR (DNA analysis). Initial and 24-hour repeat EEG’s confirmed a diffuse disturbance in cerebral function. Following six days of acyclovir treatment, there was no improvement in the patient’s neurologic status and his family members elected to withdraw his life-sustaining treatments. Postmortem Lyme IgG was confirmed on ELISA, although neither HSV 1 nor 2 were detected by PCR. After the patient’s death, WNV antibodies were detected in the CSF.

## DISCUSSION

This patient had multiple risk factors for the neuroinvasive form of WNV infection, most notably his CLL and recent treatment with ibrutinib. Earlier examples have demonstrated that WNV can persist for as long as four months post-mortem in immunocompromised hosts with humoral deficiency from B cell lymphoma.^2^ This particular patient’s clinical presentation was unique in that there was no viral symptoms or neurologic changes suggesting acute WNV infection.  Initially, he had presented with sepsis due to cellulitis.

As this patient’s hospital course continued, he developed worsening neurologic symptoms that progressed to a comatose state prompting the authors to evaluate him for a possible central nervous system infection. Specific physical exam findings included disorientation, fever, tachycardia, bilateral asymmetric weakness, hypo-reflexia, arrhythmic muscular jerking, tremor, and lack of coordination.

The authors have located other case reports of immunocompromised patients with lymphoma describing similar neurologic findings of lethargy or confusion with weakness and muscular abnormalities.^3^ The patients described in these reports were being treated with rituximab. Rituximab is a monoclonal antibody that inhibits the body’s humoral immune response by targeting the CD 20 surface protein on B cells resulting in B cell death. Although this patient did not receive rituximab, he was treated with the chemotherapeutic agent ibrutinib. Ibrutinib operates by binding to Burton’s tyrosine kinase (BTK) receptors, which are important in the signaling pathways B cell proliferation and immune response. By blocking BTK receptors, both healthy B cell and malignant B cell survival and propagation are halted.^4^ 

It has been suggested in the literature that blunted B cell immunity, whether through a disease process or chemotherapy treatment, predisposes patients to neuroinvasive forms of WNV.^4^ Given our patient’s immunocompromised state and objective findings, we believe that neuroinvasive WNV resulted in this man’s clinical changes. Although neurologic decompensation in an immunocompromised patient should certainly prompt investigation for CNS infection, WNV encephalitis was low on our differential diagnosis because neuroinvasive WNV infections are still quite rare manifestations in Michigan. Additionally, the authors were more suspicious of an alternative infectious etiology due to his cellulitis and HSV-1 positive lip ulceration.

WNV was first detected in Michigan in August 2001. By 2003, 73 of the 83 Upper and Lower Peninsula Michigan counties had been confirmed to have WNV positive birds. The most recent occurrences of WNV treated in the authors’ health system occurred in 2006 when two patients with confirmed WNV were successfully treated and discharged after one week of supportive care.^5^ Since 1999, Michigan has consistently reported 0.50 to 0.74 per 100,000 residents of neuroinvasive WNV annually.^6^ Neurologic manifestations of this type of infection are relatively uncommon and therefore not often included in the initial workup of comatose patients.^7^

Furthermore, while this patient’s MRI was remarkable for a right thalamus abnormality, this finding was nonspecific and did not aid the authors in their diagnosis. The authors have located one documented case of confirmed neuroinvasive WNV infection in a patient with CLL demonstrating bithalamic area of T2 hyperintensity.^8^ These signal thalamus changes may serve as an additional neuroinvasive WNV diagnostic indicator. However, the gold standard for diagnosis is still WNV PCR analysis of CSF.

## CONCLUSIONS

The mainstay of treatment for neuroinvasive WNV infection is supportive care. There is evidence that intravenous immunoglobins given early may offer some benefit.^9^ However, the case described in which this approach was used did not alter the disease course and ultimately the patient expired. In retrospect, it is not clear whether diagnosing neuroinvasive WNV earlier would have changed this patient’s outcome. His neurologic symptoms were atypical for most presentations of neuroinvasive WNV infection which delayed the authors’ investigation for CNS infections. This patient’s hospital course was managed in the critical care unit. He received aggressive supportive care and appropriate assistance from consulting services including neurology and infectious disease. After 11 hospital days, his neurologic status did not improve and his life-sustaining care was withdrawn.

This case illustrates how climate and geography should be considered, and ultimately influence, the differential diagnosis of patients presenting with neurological symptoms. Furthermore, as the incidence of WNV increases in Michigan, clinicians must maintain a higher index of suspicion for WNV since objective findings including radiographic and laboratory evaluation are non-specific. This is especially imperative in immunocompromised patients since WNV infection may be one piece of a complicated clinical presentation that can be easily overlooked.

### Conflict of Interest

The authors declare no conflict of interest.
